# Prolonged repeated vaccine immuno-chemotherapy induces long-term clinical responses and survival for advanced metastatic melanoma

**DOI:** 10.1186/2051-1426-2-9

**Published:** 2014-04-15

**Authors:** Brendon J Coventry, Carrie A Lilly, Peter Hersey, Antonio Michele, Richard J Bright

**Affiliations:** Discipline of Surgery, University of Adelaide, Adelaide Melanoma Unit, Royal Adelaide Hospital, Adelaide, South Australia Australia; Kolling Institute University of Sydney, New South Wales, Australia; Medical Oncology, North Adelaide Oncology, Calvary Hospital, North Adelaide, South Australia 5006 Australia

**Keywords:** Metastatic melanoma, Vaccine, Immunotherapy, Immuno-Chemotherapy, Survival, Complete response, VMCL, Repetitive vaccinations

## Abstract

**Background:**

Repetitive long-term Vaccinia Melanoma Cell Lysate (VMCL) vaccination schedules have proved clinically effective in producing Complete Responses and strong durable survivals for up to 6.1 years in a previous study of patients with advanced Stage IV and Stage IIIc melanoma. These studies were expanded to include 54 patients for further evaluation of these findings.

**Methods:**

54 patients comprising 48 Stage IV (6 M1a, 14 M1b, 28 M1c) and 6 advanced Stage III (5 IIIc; 1 IIIb) were studied using repeated intra-dermal VMCL vaccine therapy. If disease progressed, vaccine was continued together with standard chemotherapy (DTIC and/or Fotemustine). Overall survival was the primary end-point assessed, with clinical responses and toxicity recorded.

**Results:**

From vaccine commencement, median overall survival was 14 months, ranging from 4 to 121 months. Kaplan-Meier survival analysis demonstrated overall 1, 2 and 3-year survival estimates of 57%, 26% and 18.5% respectively, and overall 5-year survival of 15.4%. No appreciable toxicity was observed. Complete Responses (CR) occurred in 16.7% (9) and partial responses (PR) in 14.8% (8) of patients. Stable disease was noted in a further 25 patients (46.3%). No response to therapy was apparent in 12 patients (22.2%). The overall response rate was 31.5% (CR + PR), with clinically significant responses (CR + PR + SD) in 77.8% of patients. Strong, durable clinical responses with overall survivals ≥ 23 months occurred in 29.6% of patients treated with repeated VMCL vaccine for advanced melanoma, (+/- concurrent chemotherapy).

**Conclusions:**

Prolonged, repetitive VMCL vaccination immunotherapy appears to be a clinically effective means of generating relatively high CR rates, useful clinical responses and long-term survivals, with little toxicity, but remains notably under-explored. Successive immunomodulation might explain the results. Closer analysis of repetitive dosing is required to improve clinical response rates and survival, perhaps by optimising the timing of immunotherapy delivery.

**Trial registration:**

Australian and New Zealand Clinical Trials Registry ANZCTRN12605000425695.

**Electronic supplementary material:**

The online version of this article (doi:10.1186/2051-1426-2-9) contains supplementary material, which is available to authorized users.

## Background

Survival of patients with advanced disseminated melanoma remains poor using almost any therapeutic means currently available. In those relatively rare situations where surgery can be utilised in selected patients with metachronous or synchronous metastatic melanoma deposits, 5-year survivals of 5-35% have been reported [1–3]. Recent advances in transduction pathway inhibition with B-raf and MEK, or immunotherapy using CTLA-4 and PD-1/L1 inhibitory antibody therapies have produced some encouraging results [4–14]. However, the majority of the clinical responses have not been translating into complete responses (CR), nor long-term survivals. The currently reported best CR rates have been associated with immunotherapies such as interleukin-2 (IL-2) (5-10%), combined IL-2 and CTLA-4 inhibitor therapies (17%), vaccine therapy (18.9%), and cellular therapy (22%) [8, 15, 16]. Vaccinia Melanoma Cell Lysate (VMCL) vaccine therapy has been reported previously, and the role of the immune system, vaccines and immuno-chemotherapy have been discussed [15, 17–23]. The results of an initial pilot study using repeated doses of VMCL vaccine therapy in 37 patients with advanced surgically non-resectable stage IV/ IIIc metastatic melanoma demonstrated a CR rate of 18.9%, with strong durable survivals for up to 6.1 years, and survival rates of 40.5%, 21.6% and 10.8% at 1, 2 and 3 years respectively [15].

The primary aim of the present study was to investigate overall survival, with clinical response rates and toxicity also being evaluated, using the repetitive VMCL vaccine therapy approach, in a larger cohort of 54 patients with surgically non-resectable advanced Stage IV/ III in-transit metastatic melanoma.

## Methods

### Patients and inclusion/exclusion criteria

Informed consent was obtained before entry into the study, which was approved by the Royal Adelaide Hospital Human Research Ethics Committee and was registered with the Australian Clinical Trials Registry [ACTRN 12605000425695]. 54 stage IV/IIIc advanced melanoma patients were enrolled in these studies for the primary aim. Most patients had failed other therapies. Confidentiality of patient data was preserved. Overall quality of life was measured by assessing functional status using the standard Eastern Cooperative Oncology Group (ECOG) and Union for International Cancer Control (UICC) scores. CT scans were performed each 3 months and ophthalmology examinations (for possible melanoma associated retinopathy/iritis) each year, or as clinically indicated.

#### Inclusion criteria

Patients ≥ 18 years of age; ECOG 0-2; evaluable metastases from primary cutaneous melanoma; advanced non-surgically resectable AJCC Stage IV or Stage IIIb/c disease; tumour volume < 20 cm diameter or < 70% of organ replacement; +/- post-surgical treatment of brain metastases; voluntary informed consent.

#### Exclusion criteria

Second primary invasive cancer (not BCC, SCC of skin or resected in-situ malignancy); untreated brain metastases, extremely extensive metastatic disease, bone metastases only; high-dose oral steroid therapy; pregnant or lactating; severely atopic individuals; severe cachexia; immunodeficiency; HIV, Hepatitis B or C positive. Three patients with inoperable brain metastases were excluded. No patient was both eligible and refused to participate.

### VMCL vaccine

Vaccinia Melanoma Cell Lysate (VMCL) vaccine was manufactured using successive aliquots of a single stable culture seed lot of the allogeneic melanoma cell line MM200, which were thawed as required, briefly cultured then infected with vaccinia virus (CSL laboratories, Melbourne) to cause cell lysis. The thawed MM200 aliquots were determined to be stable over time using karyotype, western blot and antigenic analysis. Lysed cells were ultrasonicated and centrifuged to create the allogeneic cell lysate vaccine product for use as described previously [15, 17–20]. Each vaccine had a protein content of 100mcg per 0.3 ml dose, equivalent to 5 × 10^6^ cells per dose. Vaccine doses were frozen to preserve protein content at -20°C and thawed to room temperature before use. The process had been previously used successfully in a previous Australian randomised clinical trial for earlier-stage, completely resected high-risk melanoma [20].

### VMCL vaccinations

All patients received regular 2-weekly single-dose intradermal vaccinations for 6 months; then monthly for 6 months; then if stabilisation or a CR was obtained, 3-monthly thereafter. Injection sites were rotated between upper outer aspects of all 4 limbs, but avoiding any limb where lymph node dissection was performed. Previous VMCL studies using 0.3 ml of re-suspended sonicated lysate had determined safety and efficacy of this dose and schedule [15, 17–20, 24]. Occasional minimal skin reactions were noted in the pilot or previous studies, and precautionary resuscitation facilities were available (but never required) with patients being observed for 30 minutes after each vaccination.

### Chemotherapy

Melanoma disease progression during vaccine therapy indicated addition of concurrent standard chemotherapy (either dacarbazine (DTIC) 1000 mg/m^2^ at 3-weekly intervals intravenously), or fotemustine (100 mg/m^2^ weekly intravenously for 3 weeks, then 4 weekly thereafter) [25, 26]. Vaccinations were maintained at 2-weekly intervals throughout the chemotherapy period, including between doses and during breaks in chemotherapy. Occasional vaccine schedule adjustment was required to suit the chemotherapy schedule.

### Skin Delayed Type Hypersensitivity (DTH)

The 1st and the 4th VMCL vaccination doses (0.3 ml) were investigated to examine DTH responses. Each read-out was 48 hours after vaccine administration. Erythema and the induration were recorded and independently recorded in two directions perpendicular to each other. Responses at the vaccination sites > 10 mm were considered positive.

### Clinical end-points

#### Primary end-point

*Overall survival* was assessed by survival in months from the time of commencement of vaccination to the date of analysis or death of the patient.

#### Secondary end-points

(i) *Toxicity and tolerability*: local or systemic reactions were recorded.

(ii) *Tumour response rates*: Rates of Complete Response (CR), Partial Response (PR), Stable Disease (SD) and Progressive Disease (PD) were recorded using the WHO criteria [27]. Observable subcutaneous lesions were assessed using direct size measurement using calipers or a ruler, and internal metastases were assessed using CT scans at 3-monthly intervals or as clinically otherwise determined, and where appropriate using ultrasound, MRI or Positron Emission Tomographic (PET) scans. Measurements were in two directions perpendicular to each other.

### Statistical analysis

This was performed using mean and median calculations, Kaplan-Meier analysis and time series analysis with the assistance of statisticians and a mathematician (TS; NB; AC). A significance level of p < 0.05 was set for all analyses.

## Results

Data for these results were collected from enrolment of the first patient in November 2000, until data analysis of 54 patients at the end of 2010. The period from the date of vaccination commencement of the first patient to the end 2010 was 10 years and 1 month (3690 days).

### Patient characteristics

The median age of the 54-melanoma patients enrolled in the study was 66 years (range 35-97), the majority of whom had Stage IV disease. Other demographic/classification data are demonstrated in Table 1.

**Table 1:** **Characteristics for the 54 patients entered into the study**

***Parameter***	***VCML treated (n = 54)***	
***Age (Yrs)***		
Mean	61.6	
Median	66	
***Range***	35-97	
<55	18	
55- < 65	14	
65+	22	
***Gender***		
Male	32	
Female	22	
**Performance status**		
	**ECOG**	**UICC**
	0 = 51	0 = 51
	1 = 2	1 = 2
	2 = 1	2 = 1
**M classification**		
IIIb	1 (2%)	
IIIc	5 (9%)	
M1a	6 (11%)	
M1b	14 (26%)	
M1c	28 (52%)	

### Primary endpoint – overall survival

**Figure 1:**
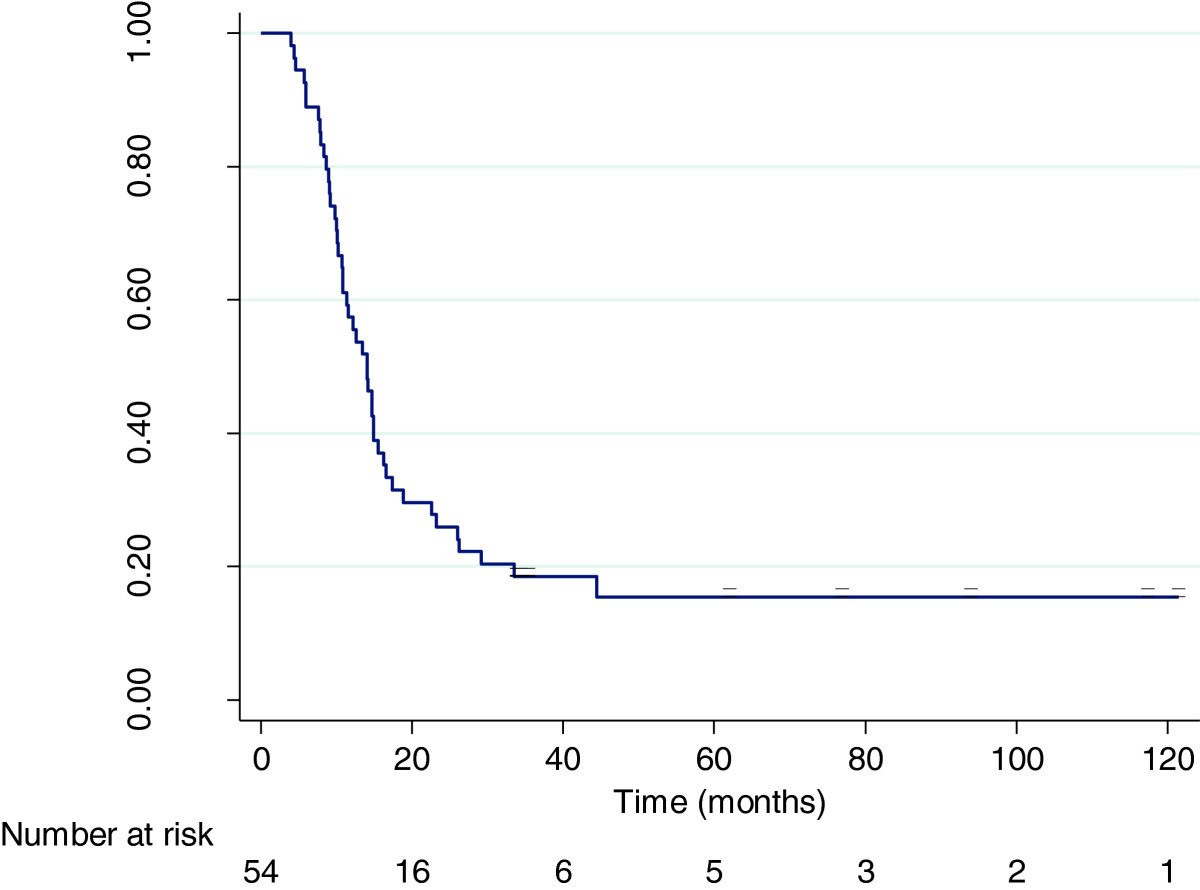
**Kaplan-Meier survival curve for VMCL Treated Patients (n = 54).**

Overall survival (to either death or the date of analysis) for all 54 patients ranged from 4 months to 121 months. Median survival was 14 months, with a mean survival of 22.5 months. A Kaplan-Meier survival analysis was performed which demonstrated overall 1, 2 and 3-year survival estimates of 57%, 26% and 18.5% respectively. The overall 5-year survival estimate was 15.4%. These are as shown in Figure 1.

**Figure 2:**
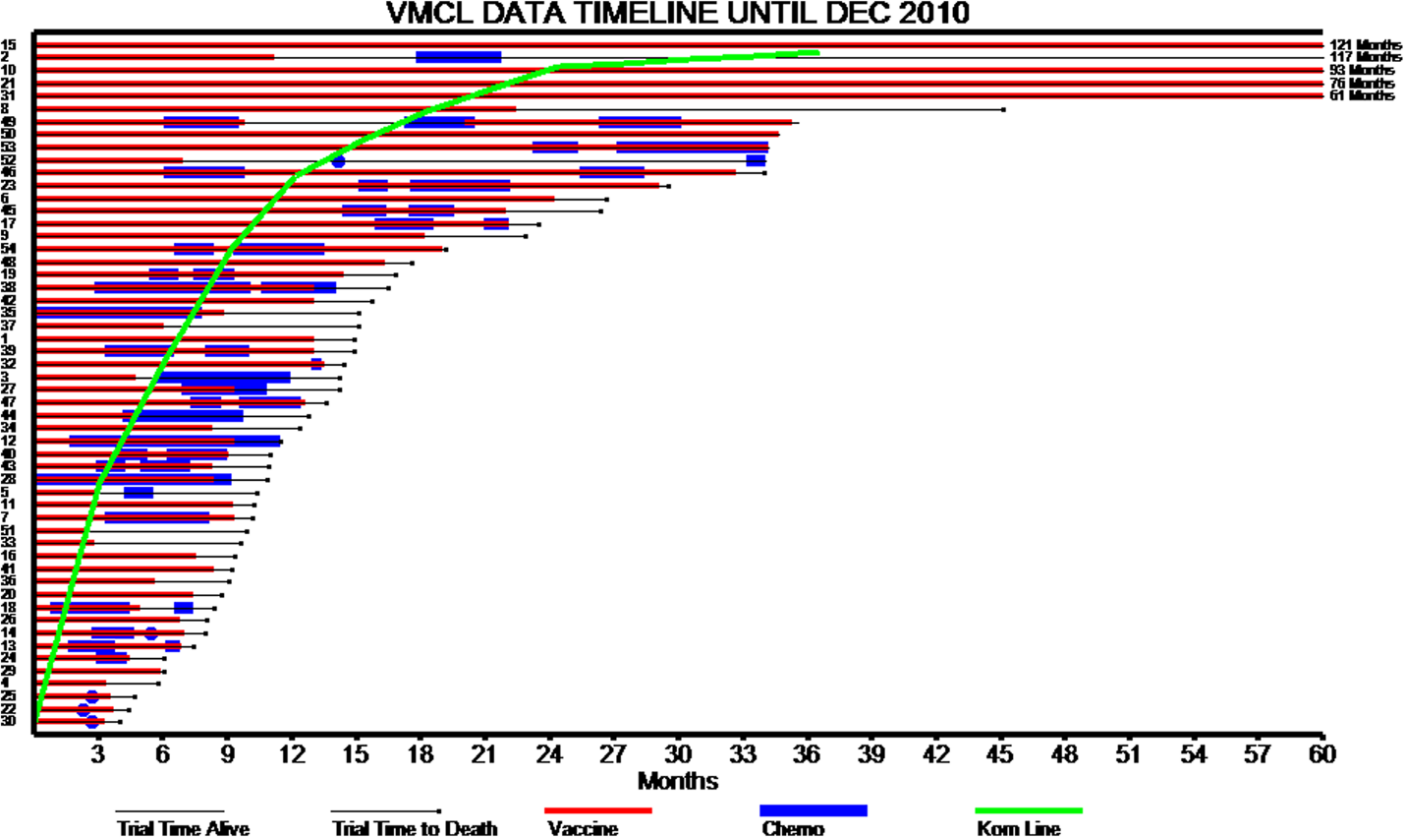
**Individual patient survival and treatment data to December 2010 (n = 54) [Korn line is survival data from Korn et al.,**[28]**]; time is to follow-up or death; vaccine is VMCL; Chemotherapy is systemic chemotherapy].**

Survival duration and treatment type patterns are also shown in Figure 2 to further illustrate patient responses.

At analysis, an observed survival of 12 months or more occurred in 55.6% or 30 of the 54 patients.

Survival greater than 23 months occurred in 29.6% (16) of the 54 patients, ranging from 1.9 years to 10.1 years. Of those 16 patients, organ +/- lymph node metastases were present in 10 patients (62.5%).

Multiple subcutaneous/cutaneous metastases alone occurred in the remaining 6 patients (37.5%). Characteristics of these 16 patients are shown in Table 2.

**Table 2:** **Clinical outcome details for 16 patients surviving ≥ 23 months including survival times (to follow-up at end 2010 or death) in months, disease sites at commencement of vaccination, treatments prior to commencement of vaccination and current status**

Survival (Months)	ID	Disease sites	Prior Tx	Status
121	015	s/c, LN	S, R	Alive, fully functional
117	002	S/C, lung, LN	S, B	Alive, fully functional
93	010	s/c	S, ILI	Alive, fully functional
76	021	s/c	S, ILI	Alive, fully functional
61	031	s/c, lung, LN	S, R	Alive, fully functional
44	008	s/c	S	Died
35	049	s/c, lung	S	AWD, fully functional
34	050	s/c, LN, brain, spleen	R, S	Alive, fully functional
34	046	s/c, lung	S	Died
33	053	s/c, lung	S	Alive, fully functional
29	023	S/C, GB, lung	S	Died
27	052	s/c, umbilical	S	Off trial/died
26	045	s/c	S	Died
24	006	s/c	S	Died
24	017	s/c, LN, lung, liver, spleen	S	Died
23	009	S/C, Bone, lung	S, R	Died

*Abbreviations*: *s/c*sub-cutaneous, *LN* lymph nodes, *GB* gall bladder, *S* surgery, *B* biological therapy, *ILI* isolated limb infusion (of chemotherapy), *R* radiotherapy, *AWD* alive with disease.

For the entire group, at the end of the survey period, 9 (17%) patients were alive and 45 (83%) had died. Melanoma was the cause of death in all 45 patients at the completion of study period. The median (mean) survival time of the 9 patients alive was 61 (67) months from vaccine commencement.

### Clinical disease responses

Complete Responses occurred in 16.7% (9) and partial response (PR) in 14.8% (8) patients. Stable disease was noted in a further 25 patients (46.3%). In 12 patients (22.2%) no response to therapy was apparent. The overall response rate was 31.5% (CR + PR), with clinically significant responses (CR + PR + SD) in 77.8% of patients. Of the complete responders, complete durable regression of all disease beyond 18 months (alive + CR) occurred in 7 patients (12.9%).

Of the 9 (16.7%) CR’s that occurred, 5 were in patients who received VMCL vaccine alone, and 4 had vaccine with systemic chemotherapy.

**Figure 3:**
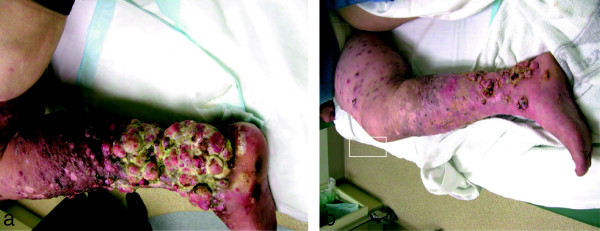
**Effects of VMCL vaccine alone on a patient treated from August 2005 (before therapy; a) to December 2005 (during therapy; b) using repetitive dosing.** She was able to walk after the therapy and the tumour size, pain, and odour decreased, with substantial improvement in self-care and walking ability. (reproduced with permission Journal of Cancer Therapy^©^[15]).

Responses were sometimes associated with quite remarkable regression of large masses of tumour as shown in Figure 3.

### Toxicity

No toxicity issues pertaining to VMCL vaccine administration alone were experienced. Any reported toxicities were related to known standard chemotherapy side effects and appeared unaffected by concomitant vaccination.

### Delayed Type Hypersensitivity (DTH)

There were no significant DTH responses observed.

### Prior clinical treatment type

All patients had some form of surgery prior to their entry into the trial. This ranged from surgery for initial diagnosis and wider excision, to that performed for regional or systemic metastatic disease control.

Prior treatments (excluding surgery) occurred in 32 patients included chemotherapy and/or radiotherapy or experimental biological therapies (6 patients: C-vax (x2 BCG-Placebo; x1 BCG±C-Vax), Heat shock protein (x1), NYESO-Iscom matrix (x1); IL-18 (x1)). 22 patients did not receive any prior therapy. From commencement on the trial, 14 patients received vaccine therapy alone, of which 8 had received no previous therapy. As part of the study, 31 patients received systemic chemotherapy in addition to the VMCL vaccine therapy.

## Discussion

The CR rates and long-term survivals using the VMCL vaccine approach are appreciable, even when compared with newer ‘targeted’ agents, indicating that prolonged, repetitive vaccination approaches require further detailed evaluation. Although several promising newer therapies have increased the armamentarium for management of advanced stage IV and stage III melanoma, notably B-raf, MEK, CTLA-4 and PD-1/PDL-1 inhibitory therapies, there has remained little progress in the development of treatments that induce complete responses and long-term survival for melanoma patients [4–14]. The reported complete response rates for B-raf therapies was 3-6%; ipilimumab, 0.9-1.5%; combined Braf/Mek, 2%; and PD-1, 1%; PDL-1, 6%; and CTLA-4/ PD-1, 9.6% [4–14]. These CR rates have unfortunately not yet translated into durable cure rates with long-term disease free 5- and 10-year survivals. A recent meta-analysis of 38 targeted therapies has sparked editorial comments concerning the efficacy and toxicity of numerous ‘targeted’ agents, questioning the selectivity due to ‘off-target’ effects and the level of clinical efficacy given their presumed specificity [29, 30].

Clearly, there is a current need for improvement in both complete response rates and survival times for patients with advanced melanoma.

The current extended study results in 54 melanoma patients compared favourably with those previously described above (for 37 patients [15]) demonstrating rates of CR 16.7%; PR 14.8%; ORR of 31.5%, SD in 46.3%, with any clinically meaningful response (CR + PR + SD) in 77.8% of patients.

The primary endpoint of survival was notable using the repetitive VMCL vaccine therapeutic approach. The Kaplan-Meier 5-year survival estimate in this study was 15.4%, which is itself significant, with the longest survivor currently alive at 10.1 years duration. The fact that 29.6% (16) of the 54 patients survived 23 months or longer is of significance also. Survival times within that group ranging from 1.9 years to 10.1 years are remarkable, indicating that survival could be considerably prolonged, compared with essentially all other treatments reported to date. Our overall survival data at 3-years using VMCL compared favorably with some recent preliminary results using pooled analysis of CTLA-4 antibody therapy, although the CR rate with VMCL therapy appeared appreciably higher than for CTLA-4 therapy [31].

The resurgence of interest in immunotherapeutic methods (e.g. IL-2, CTLA4, PD-1, PDL-1) for the treatment of metastatic melanoma and cancer in general, opens new approaches to therapeutic design through the better understanding of modulation of the immune system. We reason that successive vaccination over a prolonged period can modulate the immune system in the patient, with the existing non-resectable melanoma metastases serving as persistent sources of tumour antigen. This approach of repeatedly boosting or successively immuno-modulating appears to be capable of stimulating or ‘re-setting’ the endogenous immune response occurring in the melanoma patient. The delivery of repeated boosting signals (in the form of the VMCL vaccine) may enhance immune responses by better synchronisation and coordination of the pre-existing endogenous immune response against the tumour. Indeed, induction of allergic tolerance, or the converse, allergic sensitisation, has been well demonstrated for many years. It has been increasingly appreciated and shown that repeated small doses of an antigen can induce either tolerance or responsiveness respectively, to that same antigen/allergen. The immune system appears capable of being ‘re-educated’ to even life-threatening bee-venom or peanut allergies by repetitive small dose therapies in order to induce clinically effective tolerance. The prolonged, repetitive VMCL vaccine approach we describe, might reasonably represent a reversal of this process, to effectively induce responsiveness through breaking tolerance. Indeed, repeated release of tumour antigen from the cancer *in vivo* might be responsible for systemic tolerance so widely observed in advanced cancer patients. We have previously suggested that multiple cancer therapy approaches are able to induce immune stimulation (even CR’s), and that the timing of the delivery of the stimulus for induction of the immune response is crucial for the immune response to be driven in the correct direction for optimal synchronisation of an effector response. The corollary is that mis-timing could drive the immune response in the opposite direction to induce tolerance rather than responsiveness [32–34]. This concept of immunotherapeutic timing and synchronisation has been extended and reviewed recently in renal cell cancer therapy using IL-2 [35]. The recent observation of the oscillatory behavior of the immune response against cancer may allow better targeting of anti-cancer therapies [32, 33, 36, 37]. The resurgence of interest in immunotherapies, including vaccines, may open the way for careful immune monitoring, improved understanding of immune modulation, and perhaps better synchronisation of therapies, including combined therapies, in order to achieve improved clinical responses.

## Conclusions

Prolonged, repetitive VMCL vaccination immunotherapy appears to be a clinically effective means of generating relatively high CR rates, useful clinical responses and long-term survivals, with little toxicity. Successive immunomodulation through repetitive stimulation of the underlying endogenous immune response to the cancer might explain these results. This phenomenon remains notably under-explored. Closer analysis of repetitive dosing is required to improve clinical response rates and survival, perhaps by optimising the timing of immunotherapy delivery. Improved synchronisation of delivery of therapies with the existing immune response already occurring in the patient, might offer a gentler means of successful modulation of the immune response and, if true, would represent a major advancement in cancer control.

## Authors’ original submitted files for images

Below are the links to the authors’ original submitted files for images. Authors’ original file for figure 1 Authors’ original file for figure 2 Authors’ original file for figure 3

## References

[1] Howard JH, Thompson JF, Mozzillo N, Nieweg OE, Hoekstra HJ, Roses DF, Sondak VK, Reintgen DS, Kashani-Sabet M, Karakousis CP, Coventry BJ, Kraybill WG, Smithers BM, Elashoff R, Stern SL, Cochran AJ, Faries MB, Morton DL (2012). Metastasectomy for distant metastatic melanoma: analysis of data from the first Multicenter Selective Lymphadenectomy Trial (MSLT-I). Ann Surg Oncol.

[2] Ollila DW, Gleisner AL, Hsueh EC (2011). Rationale for complete metastasectomy in patients with stage IV metastatic melanoma. J Surg Oncol.

[3] Balch CMHA, Sober AJ, Soong SJ (2008). Cutaneous Melanoma.

[4] Flaherty KT, Puzanov I, Kim KB, Ribas A, McArthur GA, Sosman JA, O’Dwyer PJ, Lee RJ, Grippo JF, Nolop K, Chapman PB (2010). Inhibition of mutated, activated BRAF in metastatic melanoma. N Engl J Med.

[5] Flaherty KT, Robert C, Hersey P, Nathan P, Garbe C, Milhem M, Demidov LV, Hassel JC, Rutkowski P, Mohr P, Dummer R, Trefzer U, Larkin JM, Utikal J, Dreno B, Nyakas M, Middleton MR, Becker JC, Casey M, Sherman LJ, Wu FS, Ouellet D, Martin AM, Patel K, Schadendorf D, METRIC Study Group (2012). Improved survival with MEK inhibition in BRAF-mutated melanoma. N Engl J Med.

[6] Hodi FS, O’Day SJ, McDermott DF, Weber RW, Sosman JA, Haanen JB, Gonzalez R, Robert C, Schadendorf D, Hassel JC, Akerley W, van den Eertwegh AJ, Lutzky J, Lorigan P, Vaubel JM, Linette GP, Hogg D, Ottensmeier CH, Lebbé C, Peschel C, Quirt I, Clark JI, Wolchok JD, Weber JS, Tian J, Yellin MJ, Nichol GM, Hoos A, Urba WJ (2010). Improved survival with ipilimumab in patients with metastatic melanoma. N Engl J Med.

[7] Chapman PB, Hauschild A, Robert C, Haanen JB, Ascierto P, Larkin J, Dummer R, Garbe C, Testori A, Maio M, Hogg D, Lorigan P, Lebbe C, Jouary T, Schadendorf D, Ribas A, O’Day SJ, Sosman JA, Kirkwood JM, Eggermont AM, Dreno B, Nolop K, Li J, Nelson B, Hou J, Lee RJ, Flaherty KT, McArthur GA, BRIM-3 Study Group (2011). Improved survival with vemurafenib in melanoma with BRAF V600E mutation. N Engl J Med.

[8] Prieto PA, Yang JC, Sherry RM, Hughes MS, Kammula US, White DE, Levy CL, Rosenberg SA, Phan GQ (2012). CTLA-4 blockade with ipilimumab: long-term follow-up of 177 patients with metastatic melanoma. Clin Cancer Res.

[9] Robert C, Thomas L, Bondarenko I, O’Day S, DJ M, Garbe C, Lebbe C, Baurain JF, Testori A, Grob JJ, Davidson N, Richards J, Maio M, Hauschild A, Miller WH, Gascon P, Lotem M, Harmankaya K, Ibrahim R, Francis S, Chen TT, Humphrey R, Hoos A, Wolchok JD (2011). Ipilimumab plus dacarbazine for previously untreated metastatic melanoma. N Engl J Med.

[10] Atkins MB, Kunkel L, Sznol M, Rosenberg SA (2000). High-dose recombinant interleukin-2 therapy in patients with metastatic melanoma: long-term survival update. Cancer J Sci Am.

[11] Topalian SL, Hodi FS, Brahmer JR, Gettinger SN, Smith DC, McDermott DF, Powderly JD, Carvajal RD, Sosman JA, Atkins MB, Leming PD, Spigel DR, Antonia SJ, Horn L, Drake CG, Pardoll DM, Chen L, Sharfman WH, Anders RA, Taube JM, McMiller TL, Xu H, Korman AJ, Jure-Kunkel M, Agrawal S, McDonald D, Kollia GD, Gupta A, Wigginton JM, Sznol M (2012). Safety, activity, and immune correlates of anti-PD-1 antibody in cancer. N Engl J Med.

[12] Brahmer JR, Tykodi SS, Chow LQ, Hwu WJ, Topalian SL, Hwu P, Drake CG, Camacho LH, Kauh J, Odunsi K, Pitot HC, Hamid O, Bhatia S, Martins R, Eaton K, Chen S, Salay TM, Alaparthy S, Grosso JF, Korman AJ, Parker SM, Agrawal S, Goldberg SM, Pardoll DM, Gupta A, Wigginton JM (2012). Safety and activity of anti-PD-L1 antibody in patients with advanced cancer. N Engl J Med.

[13] Ott PA, Hodi FS, Robert C (2013). CTLA-4 and PD-1/PD-L1 blockade: new immunotherapeutic modalities with durable clinical benefit in melanoma patients. Clin Cancer Res.

[14] Wolchok JD, Hodi FS, Weber JS, Allison JP, Urba WJ, Robert C, O’Day SJ, Hoos A, Humphrey R, Berman DM, Lonberg N, Korman AJ (2013). Development of ipilimumab: a novel immunotherapeutic approach for the treatment of advanced melanoma. Ann N Y Acad Sci.

[15] Coventry BJ, Hersey P, Halligan A-M, Michele A (2010). Immuno-chemotherapy using repeated vaccine treatment can produce successful clinical responses in advanced metastatic melanoma. J Cancer Ther.

[16] Rosenberg SA, Yang JC, Sherry RM, Kammula US, Hughes MS, Phan GQ, Citrin DE, Restifo NP, Robbins PF, Wunderlich JR, Morton KE, Laurencot CM, Steinberg SM, White DE, Dudley ME (2011). Durable complete responses in heavily pretreated patients with metastatic melanoma using T-cell transfer immunotherapy. Clin Cancer Res.

[17] Hersey P, Mitchell MS (1992). Vaccinia viral lysates in treatment of melanoma. Biological Approaches to Cancer Treatment: Biomodulation.

[18] Hersey P (1993). Evaluation of vaccinia viral lysates as therapeutic vaccines in the treatment of melanoma. Ann N Y Acad Sci.

[19] Hersey P (2002). Advances in the non-surgical treatment of melanoma. Expert Opin Investig Drugs.

[20] Hersey P, Coates AS, McCarthy WH, Thompson JF, Sillar RW, McLeod R, Gill PG, Coventry BJ, McMullen A, Dillon H, Simes RJ (2002). Adjuvant immunotherapy of patients with high risk melanoma using vaccinia viral lysates of melanoma. results of a randomized trial. J Clin Oncol.

[21] Nowak AK, Robinson BW, Lake RA (2003). Synergy between chemotherapy and immunotherapy in the treatment of established murine solid tumors. Cancer Res.

[22] Hsueh EC, Essner R, Foshag LJ, Ollila DW, Gammon G, O’Day SJ, Boasberg PD, Stern SL, Ye X, Morton DL (2002). Prolonged survival after complete resection of disseminated melanoma and active immunotherapy with a therapeutic cancer vaccine. J Clin Oncol.

[23] Barbour AH, Coventry BJ (2003). Dendritic cell density and activation status of tumour-infiltrating lymphocytes in metastatic human melanoma: possible implications for sentinel node metastases. Melanoma Res.

[24] Hersey P (1992). Active immunotherapy with viral lysates of micrometastases following surgical removal of high risk melanoma. World J Surg.

[25] Atkins MB (2006). Cytokine-based therapy and biochemotherapy for advanced melanoma. Clin Cancer Res.

[26] Avril MF, Aamdal S, Grob JJ, Hauschild A, Mohr P, Bonerandi JJ, Weichenthal M, Neuber K, Bieber T, Gilde K, Guillem Porta V, Fra J, Bonneterre J, Saïag P, Kamanabrou D, Pehamberger H, Sufliarsky J, Gonzalez Larriba JL, Scherrer A, Menu Y (2004). Fotemustine compared with dacarbazine in patients with disseminated malignant melanoma: a phase III study. J Clin Oncol.

[27] WHO (1979). WHO handbook for reporting results of cancer treatment.

[28] Korn EL, Liu PY, Lee SJ, Chapman JA, Niedzwiecki D, Suman VJ, Moon J, Sondak VK, Atkins MB, Eisenhauer EA, Parulekar W, Markovic SN, Saxman S, Kirkwood JM (2008). Meta-analysis of phase II cooperative group trials in metastatic stage IV melanoma to determine progression-free and overall survival benchmarks for future phase II trials. J Clin Oncol.

[29] Niraula S, Seruga B, Ocana A, Shao T, Goldstein R, Tannock IF, Amir E (2012). The price we pay for progress: a meta-analysis of harms of newly approved anticancer drugs. J Clin Oncol.

[30] Editorial: First do no harm: counting the cost of chasing drug efficacy. Lancet Oncol. 2012, 13 (9): 849-10.1016/S1470-2045(12)70404-7. 10.1016/S1470-2045(12)70404-7 22935231

[31] ECC 2013 Press Release: Advanced melanoma: Pooled analysis of long-term survival with ipilimumab. Abstract no: LBA 24, “Pooled analysis of long-term survival data from phase II and phase III trials of ipilimumab in metastatic or locally advanced, unresectable melanoma”. Melanoma and skin cancer proffered papers session. http://eccamsterdam2013.ecco-org.eu/Global/News/ECC-2013-Press-Releases-EN/2013/09/Longest-follow-up-of-largest-number-of-melanoma-patients.aspx (accessed February 2014)

[32] Coventry BJ, Ashdown ML (2012). Complete clinical responses to cancer therapy caused by multiple divergent approaches: a repeating theme lost in translation. Cancer Manag Res.

[33] Coventry BJ, Ashdown ML (2012). The 20th anniversary of interleukin-2 therapy: bimodal role explaining longstanding random induction of complete clinical responses. Cancer Manag Res.

[34] Church SE, Jensen SM, Twitty CG, Bahjat K, Hu HM, Urba WJ, Fox BA (2011). Multiple vaccinations: friend or foe. Cancer J.

[35] Dutcher JP, Wiernik PH (2013). Deconstructing and reinventing the IL-2 paradigm: can alternate dosing schedules enhance tumor effect. Kidney Cancer J.

[36] Coventry BJ, Ashdown ML, Quinn MA, Markovic SN, Yatomi-Clarke SL, Robinson AP (2009). CRP identifies homeostatic immune oscillations in cancer patients: a potential treatment targeting tool?. J Transl Med.

[37] Ashdown ML, Coventry BJ (2010). A matter of time. Australas Sci.

