# Supplementary figures and images for: Prolonged repeated vaccine immuno-chemotherapy induces long-term clinical responses and survival for advanced metastatic melanoma
Source: J Immunother Cancer. 2014 Apr 15;2:9. doi: 10.1186/2051-1426-2-9 (PMC4950896; doi:10.1186/2051-1426-2-9)

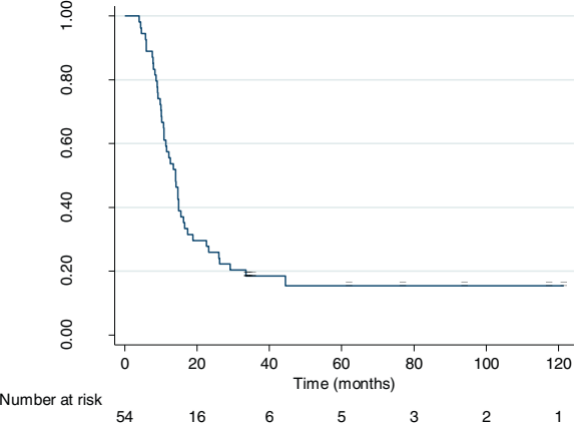

Supplement: Supplementary file 1 — Authors’ original file for figure 1 [file 40425_2013_407_MOESM1_ESM.pdf]

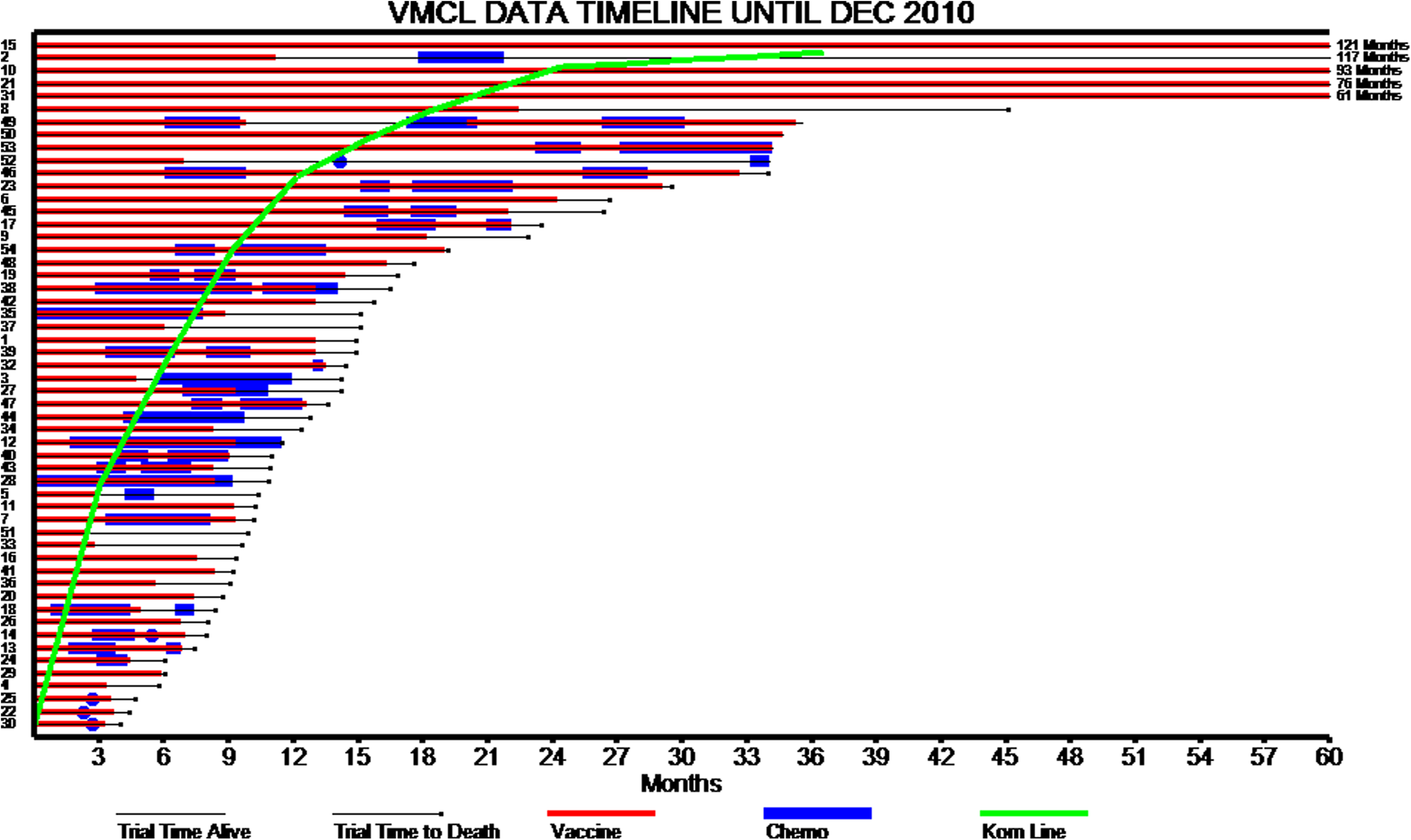

Supplement: Supplementary file 2 — Authors’ original file for figure 2 [file 40425_2013_407_MOESM2_ESM.tiff]

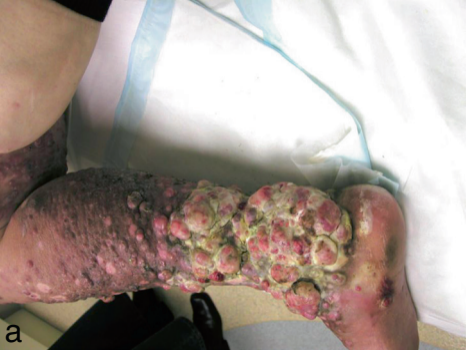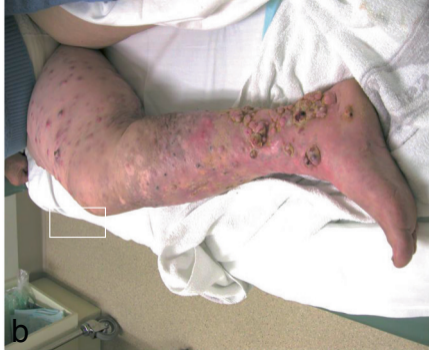

Supplement: Supplementary file 3 — Authors’ original file for figure 3 [file 40425_2013_407_MOESM3_ESM.pdf]
